# Evaluating performance of multiplex real time PCR for the diagnosis of malaria at elimination targeted low transmission settings of Ethiopia

**DOI:** 10.1186/s12936-021-04029-x

**Published:** 2022-01-06

**Authors:** Mahlet Belachew, Mistire Wolde, Desalegn Nega, Bokretsion Gidey, Legessie Negash, Ashenafi Assefa, Geremew Tasew, Adugna Woyessa, Adugna Abera

**Affiliations:** 1 Department of Medical Laboratory Sciences, College of Health Sciences, Addis Ababa University, Addis Ababa, Ethiopia; 2 Malaria and Neglected Tropical Diseases Research Team, Ethiopian Public Health Institute, P.O. Box: 1242, Addis Ababa, Ethiopia

**Keywords:** Malaria elimination, Multiplex real time PCR, Diagnostic performance

## Abstract

**Background:**

Malaria incidence has declined in Ethiopia in the past 10 years. Current malaria diagnostic tests, including light microscopy and rapid antigen-detecting diagnostic tests (RDTs) cannot reliably detect low-density infections. Studies have shown that nucleic acid amplification tests are highly sensitive and specific in detecting malaria infection. This study took place with the aim of evaluating the performance of multiplex real time PCR for the diagnosis of malaria using patient samples collected from health facilities located at malaria elimination targeted low transmission settings in Ethiopia.

**Methods:**

A health facility-based, cross-sectional survey was conducted in selected malaria sentinel sites. Malaria-suspected febrile outpatients referred to laboratory for malaria testing between December 2019 and March 2020 was enrolled into this study. Sociodemographic information and capillary blood samples were collected from the study participants and tested at spot with RDTs. Additionally, five circles of dry blood spot (DBS) samples on Whatman filter paper and thick and thin smear were prepared for molecular testing and microscopic examination, respectively. Multiplex real time PCR assay was performed at Ethiopian Public Health Institute (EPHI) malaria laboratory. The performance of multiplex real time PCR assay, microscopy and RDT for the diagnosis of malaria was compared and evaluated against each other.

**Results:**

Out of 271 blood samples, multiplex real time PCR identified 69 malaria cases as *Plasmodium falciparum* infection, 16 as *Plasmodium vivax* and 3 as mixed infections. Of the total samples, light microscopy detected 33 as *P. falciparum*, 18 as *P. vivax*, and RDT detected 43 as *P. falciparum*, 17 as *P. vivax*, and one mixed infection. Using light microscopy as reference test, the sensitivity and specificity of multiplex real time PCR were 100% (95% CI (93–100)) and 83.2% (95% CI (77.6–87.9)), respectively. Using multiplex real time PCR as a reference, light microscopy and RDT had sensitivity of 58% (95% CI 46.9–68.4) and 67% (95% CI 56.2–76.7); and 100% (95% CI 98–100) and 98.9% (95% CI 96–99.9), respectively. Substantial level of agreement was reported between microscopy and multiplex real time PCR results with kappa value of 0.65.

**Conclusions:**

Multiplex real-time PCR had an advanced performance in parasite detection and species identification on febrile patients’ samples than did microscopy and RDT in low malaria transmission settings. It is highly sensitive malaria diagnostic method that can be used in malaria elimination programme, particularly for community based epidemiological samples. Although microscopy and RDT had reduced performance when compared to multiplex real time PCR, still had an acceptable performance in diagnosis of malaria cases on patient samples at clinical facilities.

## Background

Malaria is a life-threatening disease caused by the protozoan parasite genus *Plasmodium* and transmitted by the bite of infected female *Anopheles* mosquitoes. Currently, there are five species of the genus *Plasmodium* known to cause human malaria: *Plasmodium falciparum*, *Plasmodium vivax, Plasmodium ovale*, *Plasmodium malariae*, and *Plasmodium knowlesi* [1]. Despite being preventable and treatable, malaria continues to cause significant morbidity and mortality particularly in tropics and sub-tropics area of the world [2]. In 2017, around 219 million cases and 435,000 deaths were documented globally, 92% of these cases and deaths occur in sub-Saharan Africa [3].

Malaria has been the major cause of illness and death for thousands of peoples for several years in Ethiopia; this is mainly due to variable climatic changes such as altitude and rainfall which favour the proliferation of mosquitoes [4]. Around 75% of the land of the country is malarious, in which 60% of the populations are at risk of contracting malaria infection [5]. *Plasmodium falciparum* is the predominant parasite (65%) that is known to cause the most serious infection than other species, followed by *P. vivax* (34%). Malaria transmission peaks during the harvesting season which poses a serious impact on the country’s socio-economic development [6].

Light microscopy using Giemsa-stained blood film is a primary malaria diagnostic tool and considered as gold standard for malaria parasite identification and confirmation at hospitals and health centres all over Ethiopia. In malaria-endemic rural areas rapid diagnostic tests (RDTs) are the malaria diagnostic tool [7]. Histidine rich protein 2 (HRP-2) and lactose dehydrogenase protein (LDH) are mostly used *Plasmodium*-specific protein antigen targets [8]. RDTs have improved sensitivity for detection of *P. falciparum* infection; they also require no electricity source and minimum training to perform the test, compared to microscopy. Reader bias when reading result bands and/or false negative results during hyperparasitaemia due to antigen prozone effects are limitations of RDT tests. Moreover, during mixed and low parasitaemia both microscopy and RDTs have shown reduced performance for detection of *Plasmodium* infection [9, 10].

Since 2005, malaria morbidity and mortality in Ethiopia has declined due to the implementation of malaria prevention and control programmes [11]. Encouraged by this effort, the Federal Ministry of Health plan to eliminate malaria by 2030 [12]. However, a lack of suitable tools to diagnose and treat every malaria infection and to guide surveillance are the major challenges for the national malaria elimination programme [13, 14].

To achieve the elimination of malaria requires the halting of every possible transmission of *Plasmodium* parasites within communities, which demands early and accurate diagnosis followed by prompt treatment and case management of patients [15]. The implementation of sensitive diagnostic tools is necessary in order to observe changes in prevalence, improve the quality of laboratory assessment and performance evaluation of alternative diagnostic tools [16].

The currently employed malaria diagnostic approaches throughout Ethiopia, microscopy and RDT, have poor sensitivity for detection of *Plasmodium* species in low transmission settings, which can lead to an underestimation of infection prevalence. Application of malaria RDT testing in the malaria elimination programme is threatened due to the emergence of *P. falciparum pfhrp2* and *pfhrp3* gene deletion [17]. To guide and evaluate the programme’s targeted elimination settings, highly sensitive and robust malaria diagnostic tools are required, especially for mass screening and treatment strategy [10, 14].

Nowadays, different molecular tests are in use for the detection of *Plasmodium* species. These molecular tests have been developed based on real-time quantitative PCR, with qualities of quantitative and closed systems that reduce time, labour, reagents, cost, and risk of contamination compared to conventional PCR [10]. Multiplex real-time PCR has improved the capability for detection of mixed *Plasmodium* infections and detection of *Plasmodium* species in low parasitaemia cases, with the detection limit of fewer than 2 parasites per ml and has an advantage of simultaneous detection of multiple targets in a single run to increase sensitivity and specificity of the test, compared to microscopy, RDTs and conventional PCR [14, 18].

## Methods

### Study area

The study was conducted in two malaria sentinel sites in Ethiopia: Shewa Robit health centre in the northeast and Metehara health centre in eastern Ethiopia. The healthcare facilities are located in the targeted malaria elimination areas. Ethiopian Public Health Institute (EPHI) in collaboration with Federal Ministry of Health established 25 malaria sentinel surveillance sites, representing regional malaria cover in Ethiopia. These sites are eco-epidemiologically representative and focal areas of malaria infection.

### Study design and period

A facility-based, cross-sectional study was conducted by collecting data from malaria-suspected febrile outpatients referred for malaria testing to the laboratory within the period December, 2019 to March, 2020.

### Participant selection criteria

All malaria-suspected febrile patients of any age referred to the laboratory for malaria testing were included in this study. Patients who had anti-malarial therapy in the 4 weeks before sample collection, and any critically ill patients were excluded from the study.

### Sample size calculation

Sample size was calculated using Buderer’s formula [19] as follows:

$${\text{Z}}^{{2}}_{1 - \alpha /2} \times S_{N} \times \left( {{1} - S_{N} } \right)$$

$${\text{L}}^{{2}} \times {\text{Prevalence}}$$

Z_1−α/2_ (standard normal deviate corresponding to the specified size of the critical region (α) = 1.96, SN (anticipated sensitivity) = 0.9, Prevalence = 13%, L (absolute precision desired on either side of sensitivity) = 0.1. Because no previous malaria prevalence studies using multiplex real time PCR were found, an estimated, intellectual guess was made of malaria prevalence of 13% and anticipated sensitivity of 90% (95% CI 80–100%) for *P. falciparum,* compared to conventional PCR. A total of 271 participants were enrolled into this study.

### Sampling technique

Convenient sampling technique was used on a consecutive basis to recruit study subjects referred to the laboratory for malaria testing by attending clinical staff, according to the usual standard of care, within the period December 2019 to March 2020.

### Data collection procedure

After obtaining patient consent, demographic profiles and clinical data were collected using a structured questionnaire. Using capillary blood taken from each patient, thick and thin smears were prepared for microscopy. RDT testing was performed at spot and five circles of dried blood spot (DBS) were collected on Whatman filter paper and transported by cold chain to the EPHI laboratory for molecular tests.

### Laboratory analysis

#### Rapid diagnostic test

RDT testing was performed as per manufacturer instruction using CareStart™ malaria HRP2/pLDH combo test. This test detects HRP-2 proteins specific to falciparum and pLDH. The tests were performed in the field laboratory by the health centres’ laboratory personnel as a routine malaria testing.

#### Malaria microscopy

After preparation of thick and thin blood films, slides were allowed to air-dry at room temperature, and the thin smear fixed by using absolute methanol then stored at 2–8 °C until being transported to EPHI. In EPHI parasitology laboratory, slides were stained with 10% Giemsa solution for 10 min, after being air dried both thick and thin smear were examined by an experienced laboratory technologist. An expert microscopist re-checked all positive slides, and 10% of negative slides. According to WHO malaria microscopy standard operating procedure, at least 100 high power fields (HPFs) were examined for parasite detection.

#### DNA extraction

Genomic DNA extraction was performed using QiagenQIAamp^®^ 96 DNA Blood Kit (QIAGEN Inc.) from DBS sample. Briefly, three 3-mm circles of the DBS punched out and placed into a 1.5-ml tube for processing as per manufacturer instructions. Finally, with 100 μl volume of elution buffer the DNA was eluted and stored at − 20 °C until assayed.

TaqMan fluorescence based DNA amplification and detection were performed using QuantStudio 5 Real time PCR system. For this study, the multiplex real-time PCR assay was run in two rounds. During the first run, all samples were tested by multiplexing pan-*Plasmodium*-specific and *P. falciparum*-specific primers and the second were by multiplexing *P. falciparum-* and *P. vivax*-specific primers. Briefly, each reaction mixture was prepared by mixing 2 µl of purified DNA template, 5 µl Luna Universal Probe qPCR Master Mix (New England Biolabs, Inc.), 2 µl PlasQ Primer Mix and 1 µl molecular biology grade water with a final reaction mixture volume of 10 µl. Amplifications were carried out using thermal cycling conditions: for the first PCR run 95 °C for 1 min, followed by 45 cycles of 95 °C for 15 s and 57 °C for 45 s and for the second run 95 °C for 1 min, followed by 45 cycles of 95 °C for 15 s and 53 °C for 45 s. The 3D7 DNA standard was run in each experiment and used as a positive control and nuclease free water used as a negative control. For PCR run, the positive control has 25 to 30 Ct values and all samples have Ct values < 30.0 for HsRNaseP taken as qualified run. Samples with Ct values between 12 and 40 and sigmoidal shape amplification curve were considered as positive (Table 1).

**Table 1:** Primers and probes sequences used for qPCR assays in this study

Target Gene	Oligo sequence	Fluorophores	TM °C
Pspp18S F	GCTCTTTCTTGATTTCTTGGATG		51.71
Pspp18S R	AGCAGGTTAAGATCTCGTTCG		52.4
Pspp18S Cy5	ATGGCCGTTTTTAGTTCGTG	Cyanine 5	52
PfvarATS F	CCCATACACAACCAAYTGGA		51.78
PfvarATS R	TTCGCACATATCTCTATGTCTATCT		52.76
PfvarATS FAM	TRTTCCATAAAGGT 5ʹ-3ʹ	Fluorescein	NA
Pv18S F	ACTAGGCTTTGGATGAAAGATTTTA		53.23
Pspp18S R	AACCCAAAGACTTTGATTTCTCATAA		51.65
Pv18S probe	GAATTTTCTCTTCGGAGTTTAT	Cy5-BHQ2	46
HsRNaseP F	AGATTTGGACCTGCGAGCG		53.25
HsRNaseP R	GAGCGGCTGTCTCCACAAGT		55.88
HsRNaseP_1	TTCTGACCTGAAGGCTCTGCGCG	HEX	60.62

### Data quality assurance

On-site training was given to all data collectors. All blood films, DBS samples and RDT testing were performed based on standard operating procedures. The quality of each reagent was checked before laboratory analysis was performed. Samples and reagents were stored at appropriate temperature as indicted on the manufacturer’s inserts. Internal and external quality controls were run as required during analysis, all remaining samples stored at − 20 °C, and collected data were double checked manually for completeness and consistency before data entry and analysis. Epi-Info version-7 was used to control and manage errors resulting from data entry process.

### Data analysis and interpretation

The collected data were coded, entered into Epi-Info version-7, and exported to STATA version 20 software before analysis and interpretation. Descriptive statistics was used to describe patient sociodemographic and clinical characteristics. The sensitivity, specificity, predictive values, and Kappa coefficient were estimated by comparing results from all three assays and 95% confidence interval was computed.

### Ethical considerations

This study was approved by Institutional Ethical Review Board of College of Health Sciences, Addis Ababa University and Scientific and ethical review office of EPHI (Protocol number: EPHI-IRB-219-2019). Official letter was written to sentinel sites. The confidentiality of patient-related data was maintained by avoiding possible identifiers, such as name of patient. Throughout the whole process of data collection and research work, all data were kept safe.

## Results

### Sociodemographic characteristics of study participants

From a total of 271 study participants, more than half of them were females (54.6%). The mean age was 24.12 years (± 14.83 SD), with 4 months minimum age and 90 years maximum age (Table 2).

**Table 2:** Age and gender distribution of the study participants

Age group (years)	Female, N (%)	Male, N (%)	Total
0–5	14 (5.2)	11 (4.1%)	25
6–15	23 (8.5)	20 (7.4%)	43
16–25	72 (26.6)	34 (12.5%)	106
26–40	28 (10.3)	44 (16.2%)	72
41–64	6 (2.2)	13 (4.8%)	19
≥ 65	5 (1.8)	1 (0.4)	6
Total	148 (54.6%)	123 (45.4%)	271

### Clinical characteristics of study participants

On presentation, 253 participants (93.36%) had chills and headaches. Sweating and muscle pain was diagnosed in 207 (76.38%) and 199 (73.43%), respectively.

### Laboratory results of study participants by multiplex real time PCR, microscopy and RDT

From the total 271 study participants, 26.2% (71) were enrolled from Shewa Robit and the remaining 73.8% (200) from Metahara health centre. Among 271 study participants, malaria-positive cases by microscopy, RDT and multiplex real-time PCR were 51 (19%), 61 (22.5%) and 88 (32.5%), respectively. Comparing the three methods, the positivity rate was highest for multiplex real time-PCR. The positivity rate by all three methods was increased among the age group 16–25 years old, and in male participants (Table 3).

**Table 3:** Malaria positivity by microscopy, RDT and multiplex real time PCR among study participants, December 2019 to March 2020, Ethiopia

Characteristics	Malaria positivity		
	Microscopy (n = 51)%	RDT (n = 61)%	Multiplex real time PCR (n = 88)%
Sex			
Female (n = 123)	15 (29.4)	17 (27.9)	35 (40)
Male (n = 148)	36 (70.6)	44 (72.1)	53 (60)
Age group			
0–5	5 (9.8)	5 (8.2)	7 (8)
6–15	11 (21.5)	13 (21.3)	17 (19)
16–25	23 (45)	29 (47.5)	38 (43)
26–40	10 (19.6)	11 (18)	20 (23)
41–64	2 (3.9)	3 (5)	6 (7)
≥ 65	0	0	0

### Diagnostic performance of multiplex real time PCR, microscopy and RDT in detecting malaria parasites

Using microscopy as a reference test, multiplex real-time PCR showed an overall sensitivity of 100% (95% CI 93–100), specificity of 83.2% (95% CI 77.57–87.87) and RDT sensitivity of 98.9% (95% CI 96.1–99.87) and specificity of 95% (95% CI 91.23–97.5), respectively (Table 4). Using multiplex real-time PCR as a reference, RDT had shown better sensitivity 67% (95% CI 56.2–76.7) than microscopy 58% (95% CI 46.95–68.4) but both had shown comparable specificity for the detection of *Plasmodium* infection (Table 4).

**Table 4:** Performance evaluation of three methods overall and among the study *Plasmodium falciparum* cases, from December 2019 to March 2020, Ethiopia

Characteristics	Sensitivity (CI 95%)	Specificity (CI 95%)	PPV (CI 95%)	NPV (CI 95%)
Multiplex real-time PCR vs microscopy				
Overall	100 (93–100)	83.2 (77.6–87.9)	57.9 (47–68.4)	100 (98–100)
* P. falciparum*	100 (89–100)	83.6 (78- 88)	45.83 (34–58)	100 (98.2–100)
RDT vs microscopy				
Overall	98 (89.6–99.9)	95 (91–97.5)	82 (70–0.6)	99.5 (97–100)
* P. falciparum*	100 (89–100)	95.4 (91.9–7.7)	75 (59.7–86.8)	100 (98.4–100)
Microscopy vs multiplex real-time PCR				
Overall	58 (46.9–68.4)	100 (98–100)	100 (93–100)	83.2 (77.6–87.9)
* P. falciparum*	45.8 (34–58)	100 (98.2–100)	100 (89–100)	83.6 (78.3–88.1)
RDT vs multiplex real-time PCR				
Overall	67 (56.21–76.7)	98.9 (96–99.9)	96.7(88.7–9.6)	86.2 (80.8–90.6)
* P. falciparum*	56.9 (44.7–68.6)	98.5 (95.7–9.7)	93.2 (81.3–8.6)	86.3 (81.2–90.5)

Multiplex real-time PCR had shown sensitivity and specificity of 100% and 83.61% for the identification of *P. falciparum* when microscopy was used as a reference test. RDT showed sensitivity and specificity of 100% and 95.38% for *P. falciparum* identification when microscopy was used as a reference test (Table 4). The numbers of *P. vivax* (16) identified by the three methods were not statistically sufficient to compute performance, and were omitted from the analysis.

### Result agreement between microscopy, RDT and multiplex real-time PCR

All samples that tested positive by microscopy were positive by multiplex real-time PCR. Additionally, 37 samples, that had missed microscopy testing, tested positive by multiplex-real time PCR. Except for two samples, all RDT-positive samples were also positive by multiplex real-time PCR and multiplex real-time PCR detected 29 samples missed by RDT.

Three mixed infections (*P. falciparum* and *P. vivax*) were detected by multiplex real-time PCR, one by RDT, none by microscopy. There was little difference among the three methods in detecting *P. vivax* (RDT: 17; microscopy: 18; multiplex PCR: 16). However, significantly more *P. falciparum* cases were detected by multiplex real-time PCR than RDT and microscopy (69 vs 43; 69 vs 33, respectively).

A substantial level of agreement (% of agreement 86.35) was reported between microscopy and multiplex real-time PCR with Kappa value of 0.65. There was an observed agreement of 88.56 (Kappa: 0.72) between RDT and multiplex real-time PCR; almost perfect agreement was reported between microscopy and RDT test results (Kappa value = 0.84) (Table 5).

**Table 5:** Percentage agreements in identification of *Plasmodium spp*. between microscopy, RDT and multiplex real-time PCR

Microscopy	Multiplex real time PCR					Percentage agreement (%)	Kappa value
	*P. falciparum*	*P. vivax*	Mixed	Negative	Total		
*P. falciparum*	33	0	0	0	33	86.35	0.65
*P. vivax*	1	14	3	0	18		
Mixed	0	0	0	0	0		
Negative	35	2	0	183	220		
Total	69	16	3	183	271		
RDTs result						88.56	0.72
* P. falciparum*	40	1	0	2	43		
* P. vivax*	2	14	1	0	17		
Mixed	0	0	1	0	1		
Negative	27	1	1	181	210		
Total	69	16	3	183	271		

RDT result	Microscopy						
*P. falciparum*	33	2	0	8	43	94.46	0.84
*P. vivax*	0	14	0	3	17		
Mixed	0	1	0	0	1		
Negative	0	1	0	209	210		
Total	33	18	0	220	271		

## Discussion

Microscopy and RDTs are a widely applicable malaria diagnostic tool and help achieve malaria control goals. However, malaria elimination requires more sensitive detection tools to halt transmission [14]. In this study, multiplex real-time PCR was found to have excellent sensitivity of 100% (95% CI 93–100) and better specificity of 83.2% (95% CI 77.57–87.87) compared to microscopy. A similar study from Toronto, Canada, using multiplex real-time PCR reported comparable sensitivity of 99.4% [18]. However, the specificity reported in this study was lower than microscopy (100%) and RDT (98.5%). This may be interpreted as microscopy and RDT having more false negative results compared to multiplex real-time PCR test. This in turn has an implication for transmission interruption, the ultimate goal of malaria elimination programmes.

The positivity rate reported in this study was highest for multiplex real-time PCR than the two conventional methods (Table 3). Multiplex real-time PCR detects all tests positive by microscopy and 97% of RDT positive *Plasmodium* infections. The positivity rate by all three diagnostic methods increased among younger age groups and decreased or become zero in older age groups, these findings are in line with a study conducted in West Arsi Zone, Ethiopia, that the overall malaria positivity rate by molecular testing was significantly higher than microscopy and RDT and the positivity rate among younger age groups was highest when determined by microscopy, RDT and molecular tests [20].

In this study, both microscopy and RDT missed significant number of cases compared with real-time PCR. A similar study was reported from Zanzibar, in which RDT missed a high proportion of malaria cases compared with PCR [21]. This showed that in targeted malaria elimination settings highly sensitive diagnostic tools that can detect all *Plasmodium* infection are required. Multiplex real-time PCR may be an alternative diagnostic tool for epidemiological studies and elimination verification in malaria elimination settings.

Conventional molecular tests use multi-stage procedures to detect a single parasite species, which is labour intensive, time consuming and prone to contamination [22]. However, multiplex real-time PCR has the advantage of simultaneous detection of multiple *Plasmodium* species in a single reaction. In this study, all microscopy-identified *P. falciparum* samples tested positive for *P. falciparum* by multiplex real-time PCR. From 18 *P. vivax*-positive samples by microscopy, multiplex real-time PCR identified 14 as *P. vivax* and three as mixed infections. Microscopy misidentified one *P. falciparum* sample as *P. vivax*, which was tested positive for *P. falciparum* by multiplex real-time PCR. This discordant result might be explained by the fact that the microscopy test quality is mainly influenced by staining quality, microscopist skill and parasitaemia [22]. A study conducted in southern Ethiopia was among several studies that revealed microscopy testing had lower sensitivity for species identification compared to molecular testing, which and lead to missed treatment of patients and to severe malaria. In this study, 14 cases that were microscopically diagnosed as *P. vivax* were found positive for *P. falciparum* when re-tested by nested PCR [23].

PCR has been considered as a molecular tool for *Plasmodium* detection and species identification. In addition to the detection of low parasitaemia and speciation, studies have shown that this technique is robust in identifying mixed infection that is often undetected and under-reported by RDT and microscopy assays. Detection of mixed infections provides accurate information for patient treatment, and for epidemiological studies regarding malaria transmission [24–26]. In the present study, among three mixed infections identified by multiplex real-time PCR, RDT detected one and the rest two as *P. vivax*. However, microscopy detected no mixed infections and all three mixed infections detected by multiplex PCR were identified as *P. vivax*. This may be due to very low level of parasitaemia of the co-infecting species during mixed infections. Likewise, in a study in Israel, from 10 mixed infections identified by real-time PCR, only one was identified by microscopy and RDT testing. In this study, real-time PCR correctly identified 81 malaria-positive cases, which were misidentified by microscopy and RDT [27]. In another study conducted in Switzerland to evaluate microscopy and multiplex real-time PCR, multiplex qPCR assay correctly identified the species and mixed infections with low levels of parasitaemia; in this study 71% of mixed infections were misdiagnosed by microscopy [28]. A study to determine the prevalence of mixed infection using real-time PCR in northern Ethiopia from 168 samples, found the prevalence of mixed infections were 1.8% by microscopy and 12.5% by real-time PCR [24]. As proven by results from these studies, multiplex real-time PCR has the most notable advantage of higher sensitivity to detect mixed infections and to identify species of malaria parasites accurately. It is the ideal malaria diagnostic method for countries such as Ethiopia, where *P. falciparum* and *P. vivax* are co-endemic species, unlike most African countries where *P. vivax* has low or nil endemicity [11].

The RDT test and microscopy had shown lower performance compared to multiplex real-time PCR in the current study. However, results from both assays show almost perfect agreement. One sample was tested negative by RDT and was positive for *P. vivax* by both PCR and microscopy. A false negative result might be associated with limitations of pLDH-based tests, and these tests had decreased sensitivity at low parasitaemia and performance of detection can be more affected by storage and transportation conditions than HRP-2 based tests [29]. Another explanation might be the prozone effect of hyper parasitaemia which leads to false negative results in RDT testing [8].

In the current study, RDT showed a sensitivity and specificity of 67% and 100%, respectively compared to multiplex real-time PCR. Similar results was reported from a large-scale study conducted to evaluate the performance of serological and qPCR in Brazil, with sensitivity and specificity of 69.56% and 100%, respectively [30]. The current study showed almost similar sensitivity in evaluating the performance of qPCR and RDT using microscopy as reference test for the diagnosis of malaria in returnees from endemic areas [18].

Although identifying malaria to species level has a crucial impact on patient management and transmission interruption, RDT testing is incapable of detecting non-falciparum malaria [9]. In the current study, the sensitivity of RDT for *P. falciparum* was 100% compared to microscopy as reference test. This was higher than the sensitivity found by Feleke et al. (94.4%) and Moges et al. (92.9%) using similar RDT format [31, 32].

Among the total febrile study participants in this study, a larger proportion of positive cases were identified by multiplex real-time PCR than by RDT and microscopy. However, the study was conducted in non-endemic settings comparing the three methods, microscopy and RDT missed fewer cases, reported by Nijhuis et al*.* [33]. This might be explained by the fact that the prevalence of sub-microscopic malaria cases increases in endemic settings than in non-endemic settings.

The negative predictive value of multiplex real-time PCR was found to be 100% using microscopy as a reference test. This means that the multiplex real-time PCR is good in ruling out malaria. *Plasmodium* infection could be ruled out with high certainty if individuals test negative using multiplex real-time PCR. This quality of PCR makes it an ideal diagnostic tool to be used in malaria elimination, rather than RDT and microscopy which were found to have high positive predictive value and low negative predictive value, in the current study. RDT and microscopy increases the probability of missing *Plasmodium* infection, which has negative impact on transmission interruption in elimination settings, and potentially to contribute to ongoing transmission [34].

Multiplex real-time PCR identified 69 malaria cases as *P. falciparum* infection, 16 as *P. vivax* and three as mixed infections from a total of 271 symptomatic malaria-suspected patients in the current study. Of these malaria cases, large numbers of *P. falciparum* were missed by both RDT and microscopy testing. However, all three methods showed perfect agreement in *P. vivax* species identification. This is probably due to in the case of *P. vivax* infection all developmental stages of parasites are found in the peripheral blood circulation that increases parasitic density in symptomatic patients compared to *P. falciparum* infection in which the parasite causes cyto-adherence and sequestration of infected erythrocytes which result in reduced parasite densities from peripheral blood [35]. Using microscopy as reference test, multiplex real-time PCR showed excellent sensitivity for *P. falciparum* (100%) identification, which is closely related to the finding of a study in Bangladesh on clinically suspected-malaria patients where the sensitivity of real-time PCR for *P. falciparum* using microscopy as a gold standard was 97.1% [36].

Substantial numbers of *P. falciparum* infection was detected by multiplex real-time PCR than microscopy and RDT methods. This might be explained as follows: in this study varATS *Plasmodium* gene was used for *P. falciparum* detection which is a more highly sensitive PCR primer for malaria than 18S rRNA-based PCR, which was also used in the current study for the detection of *P. vivax* [37]. Another reason may be due to the biology of the parasite which has a tendency to sequestrate in the organs during its life cycle and cannot be detected by RDT and microscopy [35].

## Conclusions

Multiplex real-time PCR is the most sensitive malaria diagnostic method that can be used in malaria elimination programmes. It had a more advanced performance in species identification and mixed infection detection than microscopy and RDT in low malaria transmission settings and showed better performance in detection of *Plasmodium* infection among febrile patients. This assay can be used for epidemiological and community-based prevalence studies and for verification of elimination in areas where malaria elimination is launched. However, microscopy and RDT still have an acceptable performance to be used as a malaria diagnostic tool in health facilities for patient treatment due their affordability and easy performance diagnostic methods.

## Data Availability

The dataset and materials used for the study are kept in a safe place in EPHI data management center.

## Abbreviations

DBS: Dried blood spot
EDTA: Ethylene diamine tetra acetic acid
EPHI: Ethiopian Public Health Institute
FMOH: Federal Ministry of Health
HPFs: High power fields
HRP-2: Histidine rich protein-2
LDH: Lactose dehydrogenase
MIS: Malaria Indicator Survey
NMSP: National Malaria Strategic Plan
NPV: Negative predictive value
PCR: Polymerase chain reaction
PPV: Positive predictive value
RDT: Rapid diagnostic test
WHO: World Health Organization
